# Review of “*Ecology and prevention of Lyme borreliosis*” edited by Marieta A. H. Braks, Sipke E. van Wieren, Willem Takken and Hein Sprong

**DOI:** 10.1186/s13071-018-2675-1

**Published:** 2018-03-06

**Authors:** Nicholas H. Ogden

**Affiliations:** Public Health Risk Sciences Division, National Microbiology Laboratory, Public Health Agency of Canada, Saint-Hyacinthe, Québec J2S 2M2 Canada

## Abstract

Marieta AH Braks, Sipke E van Wieren, Willem Takken and Hein Sprong, Editors

*Ecology and prevention of Lyme borreliosis.* In: *Ecology and control of vector-borne diseases Volume 4*

Wageningen: Wageningen Academic Publishers; 2016.

462 pages, ISBN 1875-0699.

## Review

This very wide-ranging book is a valuable resource for those beginning to study Lyme disease, be that from the perspective of medical practitioner, public health professional, epidemiologist, ecologist, microbiologist or entomologist. The introductory section eloquently identifies Lyme disease as a One Health issue requiring a transdisciplinary approach to understanding when and where disease risk occurs, and to preventing Lyme disease by either reducing risk levels in the environment, or by reducing exposure of the human population and their companion animals.

The book comprises chapters by over 40 predominantly European authors that are mostly narrative reviews, although some chapters contain original research. A panel of reviewers is named at the end of the book suggesting that both reviews and original research have been peer reviewed.

The first half of the book covers the fundamentals in extensive chapters on the ecology of Lyme disease. It includes a chapter on what determines tick vector densities in broad terms, which covers the biology of the tick *Ixodes ricinus* (the main European vector of Lyme disease) and broad environmental and host-related determinants of tick density. Later in the book there is also more detailed exploration of climate/weather variability in impacting interannual variations in tick densities. There are several chapters on the ecology of transmission cycles of the bacterial agent of Lyme disease *Borrelia burgdorferi* (*sensu lato*). These explore impacts of reservoir host species and communities (including consideration of the dilution effect), and seasonal phenology of tick activity, on infection prevalence in ticks, and on the frequencies with which species and strains of *B. burgdorferi* (*s.l.*) occur in nature. This section is western-Europe centric (and particularly Netherlands-centric, where the majority of authors are based) although reference is made to the ecology of Lyme disease in other parts of the world. Consequently, it is of most relevance as a reference resource for readers whose sphere of professional activity is in this geographical region.

Other parts of the book have more clearly international interest, even if the studies described were located in Europe. These include an introductory section that provides a useful and comprehensive description of what Lyme disease is in humans, and how it is diagnosed, in Europe and North America. The second part of the book covers many aspects of risk management, and this certainly has international appeal. This section includes chapters that detail the utility of modelling to synthesise knowledge on Lyme disease hazards and risk using a range of methods and for a range of purposes in decision-making. Chapters on risk mapping, at a range of spatial scales, describe their construction and use in Lyme disease risk management in terms of surveillance, prevention and control. For those responsible for responding to Lyme disease risk who are not familiar with modelling and risk mapping, these chapters provide a very useful and comprehensive starting point.

Chapters on prevention are also relevant to all, and this is a strength of the book. These chapters cover methods of prevention for people in general, occupational risk in particular, and in prevention of infection in pet dogs. There are extensive reviews on current methods of control (and their environmental impacts) by acaricides, tick exposure reduction in parks and forests by management of public access and pathways, and personal prevention methods. Innovative, proactive risk communication and health promotion methods are explored as well as possible novel methods of control such as biocontrol (e.g. the use of entomopathogenic fungi as environmentally friendly acaricides), anti-tick vaccines and the use of acaricide-treated livestock as a means of sweeping up ticks and diluting *B. burgdorferi* (*s.l.*) transmission in some environments. There is also an interesting section on the impact of greening cities on urban Lyme disease risk.

The book has some weaknesses. For example, other than being recognised as an important activity, surveillance is not explicitly covered. There is quite a lot of overlap and repetition amongst some of the chapters, and there is also speculation in some chapters, which in some cases is based on unpublished data. Both are an almost inevitable consequence of the chapters tending to have the form of stand-alone works rather components of a collective work. There is also some misuse of terminology (e.g. in use of the word “chronic”) and quite a lot of typographic and grammatical errors. These do not detract, however, from the overall utility of this book. It may not be the last word on Lyme disease for everyone, but it is a very good starting point and should be an addition to the library of anyone involved in research into, or management of, Lyme disease.

